# Parenting styles and preschool children's development: from network analysis perspective

**DOI:** 10.3389/fpsyg.2025.1624317

**Published:** 2025-07-11

**Authors:** Jiahao Han, Zhixiong Yan

**Affiliations:** ^1^Guangxi Education Modernization and Quality Monitoring Research Center, Nanning Normal University, Nanning, China; ^2^Laboratory of Cognitive Neuroscience and Education, School of Education Science, Nanning Normal University, Nanning, China

**Keywords:** preschool children, physical and mental development, parenting styles, LASSO, network analysis

## Abstract

Preschool children's physical and mental development forms a critical foundation for lifelong growth, with parenting styles playing a pivotal role. Previous research has primarily examined broad associations between parenting styles and general development, leaving gaps in understanding specific subdomain connections. This study investigates the intrinsic relationships and age-related dynamics between six representative parenting styles (Humiliation vs. Respect, Rejection vs. Acceptance, Punishment vs. Motivation, Dictatorship vs. Democracy, Indulgence vs. Control, and Rudeness vs. Protection) and five key developmental domains (Cognition, Emotion, Language, Art, and Body) in a large sample of preschool children. Using network analysis, we evaluated data from 6,394 Chinese parents who completed the *Preschool Development Scale* (PDS) and *Parenting Style Scale* (PRSS). Networks were constructed via Extended Bayesian Information Criterion for Graphical Lasso (EBICGLasso), with centrality, bridge analysis, and age-group comparisons (3-, 4-, and 5-year-olds) conducted. Results revealed Motivation (parenting) and Emotion (development) as the strongest Bridge Expected Influence (BEI) nodes. The Motivation-Cognition connection was the most robust across the sample. Age-specific analyses showed distinct bridge patterns: Motivation-Art dominated at age 3, Acceptance-Art at age 4, and Respect-Language at age 5. The strongest cross-cluster link shifted from Motivation-Cognition (ages 3–4) to Respect-Language (age 5). The network invariance test confirmed significant structural differences between all age groups (*M* > 0.119, *p* < 0.002). These findings highlight Motivation and Emotion as core bridges between parenting and development, and Motivation–Cognition–Emotion pathway serves as a potential theoretical model that offers explanatory value. Notably, parenting-development connections evolved from direct (Motivation-Art) to indirect (Respect-Language) associations with age. This study advances the traditional focus on global effects by revealing nuanced, age-specific linkages, underscoring the importance of tailored parenting strategies to foster preschool children's development.

## 1 Introduction

Preschool children's physical and psychological development plays a critical role in educational achievement, ontogenetic progression, and lifelong health (Polanczyk et al., 2015; Yazar and Tuzgöl Dost, 2024). Psychological development encompasses cognition, emotion, and language (Graziano and Hart, 2016), which are essential for learning, imitation, and self-expression as children mature (Kalland and Linnavalli, 2023). Physical development, including motor and artistic abilities, interacts with psychological growth to shape socialization (Eime et al., 2013). Research consistently highlights parenting styles as a key determinant of these developmental trajectories (Baumrind, 2013, 1971).

Baumrind (2013) framework classifies parenting into four primary styles: authoritative, authoritarian, permissive, and neglectful, each differentially influencing development. For instance, meta-analytic evidence links authoritative parenting to enhanced academic performance and language skills in preschoolers (Madigan et al., 2019). Cognitive neuroscience studies further associate this style with increased theta wave activity (Hofstee et al., 2022) and amygdala volume expansion (Zhong et al., 2024), underpinning social-cognitive growth. Conversely, neglectful parenting predicts heightened error-related negativity (Meyer et al., 2015) and greater risks of anxiety and depression (Sanders et al., 2015). Notably, urbanization in developing countries like China has shifted parenting approaches from punitive control toward motivational and democratic strategies (Li and Xie, 2017). Despite these advances, existing research predominantly examines broad associations between parenting styles and global developmental outcomes (e.g., academic achievement or social competence), leaving a gap in understanding how specific parenting strategies influence particular developmental domains in early childhood. Addressing this gap is theoretically significant as it allows for a more nuanced comprehension of the mechanisms through which distinct parenting behaviors impact discrete aspects of child development, thereby refining developmental models and parenting theory. Practically, filling this gap can guide parents in tailoring their parenting approaches to better align with their child's developmental needs; for example, for children with weaker language skills, a parenting style emphasizing respect may be more effective than encouragement (Doh et al., 2016).

Cognitive and emotional development, along with parenting motivation, has received significant attention in developmental research. Lazarus (1991) posited that children's cognitive processes serve as a driving force behind emotion development. Expanding on this, the achievement emotion theory suggests that children interpret environmental stimuli—such as parental motivation, disciplinary actions, or classroom experiences—through cognitive appraisal, which then elicits positive or negative emotional responses (Pekrun, 2006). Empirical studies consistently support this framework, demonstrating that parental motivation fosters cognitive and emotional development in children (Yazar and Tuzgöl Dost, 2024; Luo et al., 2021). However, this finding has yet to be substantiated in large-scale preschool populations. Graziano et al. (2007) demonstrated that the interplay between teacher-child relationships and emotional competence jointly predicts cognitive development in five-year-olds, suggesting the applicability of this framework to early childhood contexts. This raises the question: can parents, as primary educators, similarly activate such processes? To explore this, current study aims to (1) delineate the connections and age-related dynamics between parenting styles and individual developments, and (2) validate the potential “motivation–cognition–emotion” pathway. By clarifying these mechanisms, our findings could inform strategies to optimize parenting practices and enhance preschool children's physical and psychological wellbeing.

Physical and mental development dynamically reshape the connections between parenting styles and preschool children's developmental outcomes (Frosch et al., 2021). Age serves as a critical determinant in this process, driving both biological and psychological maturation. Between ages 3-5, children experience significant growth in the surface area of their frontal and parietal lobes (Brown and Jernigan, 2012), leading to rapid, nonlinear advances in socialization. This neurological development enables children to progress from passive recipients of parenting to increasingly active self-regulators (Montroy et al., 2016). Concurrently, parenting strategies evolve from immediate responses (e.g., motivation or punishment) toward more sustained psychological engagement (e.g., acceptance, respect) (Morawska et al., 2019). These developmental shifts necessitate adaptive parenting approaches. Research demonstrates that authoritarian parenting may benefit physical and academic outcomes in early childhood but becomes counterproductive in adolescence (Singh et al., 1995). Similarly, permissive parenting shows limited effectiveness in early years but gains relevance as children mature (Hindin, 2005). Such findings underscore the ontogenetic nature of parenting-child dynamics (Gracia, 2014). However, the specific age-related variations in how parenting styles influence preschool children's development remain insufficiently examined. By comparing 3- to 5-year-olds, this study investigates age-related transitions in parenting-development connections, offering crucial insights into age-sensitive caregiving strategies.

Network analysis is useful for datasets with multiple variables (Borsboom, 2017), and using this method could shed light on complex interactions among parenting styles and preschool children's development. Departing from traditional approaches that rely on latent variable models, this method directly examines observable subdomain variables and their interconnections. In network models, psychological constructs are operationalized as interconnected nodes (representing variables) and edges (reflecting their dynamic relationships), elucidating both intra- and inter-network interactions (van Bork et al., 2021). This approach is particularly valuable for studying how changes in one domain (e.g., parenting styles) may propagate to another (e.g., developmental outcomes), capturing the system's interconnected nature. Key advantages of network analysis include its ability to: (1) identify central nodes that exert disproportionate influence within a network through centrality analysis (Epskamp et al., 2018); (2) detect bridge nodes that connect distinct clusters, serving as crucial transmission points for cross-domain effects (Valente and Fujimoto, 2010); (3) facilitate direct network comparisons across groups (Dong et al., 2025). The connections between these bridge nodes are especially informative, as they may reveal fundamental mechanisms linking parenting practices to children's developmental outcomes.

In this study, we employ network analysis to construct and compare age-specific networks for children aged 3–5 years. This approach enables us to track developmental changes in node importance and connection patterns, and to derive targeted intervention strategies tailored to children's distinct developmental stages. By examining both network structures and their age-related variations, we aim to provide a nuanced understanding of how parenting strategies interact with developmental processes during this critical period.

## 2 Materials and methods

### 2.1 Participants

We recruited guardians of 3- to 5-year-old children from 2,146 kindergartens across Guangxi autonomous region of China, representing a sampling ratio of 1:100 relatives to the total preschool population in the region. Each participating kindergarten appointed a trained coordinator who was thoroughly briefed on the study objectives and assessment protocols. Following guardians' consent, participants were invited to complete an online survey within 2-day window, evaluating children's physical and mental development as well as parenting styles. To ensure high-quality data, we implemented response time validation, requiring each survey item to be completed within 10 to 40 s, with the entire assessment capped at ~50 min. Collected demographic data included children's gender, age, residential location, and kindergarten type (public or private).

This study received ethical approval from the Ethics Committee of Nanning Normal University (Approval No. JK2022031) and complied with local legislative and ethical standards. All participating guardians provided informed consent prior to data collection. The anonymized raw dataset is publicly accessible via the Science Data Bank (ScienceDB, https://www.scidb.cn/en/s/nQnaUf).

### 2.2 Measure

#### 2.2.1 Preschool development scale

The Preschool Development Scale (PDS) was developed by Feng Tingyong, based on *Guidelines for Learning and Development of Children Aged 3–6* and the *Kindergarten Education Guidelines* (Chen and Yu, 2020). The scale has been empirically validated in the Chinese context and comprises three age-specific versions for children aged 3, 4, and 5 (or older). The PDS assesses five key developmental dimensions: Cognition and Inquiry (Cognition), Social and Emotional Development (Emotion), Language and Communication (Language), Aesthetic Awareness and Expression (Art), and Health and Physical Fitness (Body). Each item was rated on a 5-point Likert scale (1 = strongly disagree, 5 = strongly agree), with higher scores indicating more advanced developmental levels. The scale demonstrated strong internal consistency, with a Cronbach's alpha coefficient of 0.91, supporting its reliability for measuring preschoolers' development.

#### 2.2.2 Parental rearing style scale (parent version)

The Parental Rearing Style Scale (PRSS) is a Chinese adaptation of the *Egna Minnen av Barndoms Uppfostran* (EMBU) scale (Hulbert, 2003), developed by Jin Mingqi and validated in the Chinese context (Jin and Liu, 2011). The scale assesses parenting behaviors through statements such as “when my child performs poorly in school, I criticize or punish them.” Responses were recorded on a 5-point Likert scale (1 = strongly disagree, 5 = strongly agree), with higher scores indicating a stronger tendency toward the right-side attributes of each parenting dimension. The PRSS measures six parenting style factors: Humiliation vs. Respect (Respect), Rejection vs. Acceptance (Acceptance), Punishment vs. Motivation (Motivation), Dictatorship vs. Democracy (Democracy), Indulgence (Laxness) vs. Control (Control), and Rudeness vs. Protection (Protection). Each factor consists of 10 items, yielding a total of 60 items. The scale demonstrated acceptable internal consistency, with a Cronbach's alpha coefficient of 0.72.

### 2.3 Statistics analysis

All statistical analyses were conducted using the open-source software Jamovi 2.4.3, including descriptive statistics, correlation analysis, and ANOVA. To examine the parenting-development relationship in preschool children, we constructed a partial correlation network using the Extended Bayesian Information Criterion for Graphical Lasso (EBIC-GLasso) method (Zhao et al., 2012; Epskamp and Fried, 2018). This approach applies the GLasso algorithm to estimate penalized regression coefficients, effectively reducing spurious edges while using the EBIC criterion to prevent overfitting. The EBIC-GLasso method is particularly robust for large-sample networks (*n* > 250), offering high sensitivity and specificity in detecting true associations (Jones et al., 2018). In the resulting network: nodes indicate variables (e.g., parenting styles, developmental dimensions). Edges represent partial correlations between nodes, with thickness reflecting the strength of these associations. Node predictability (the proportion of variance explained by its neighboring nodes) was estimated using a regression-based approach (Haslbeck and Waldorp, 2020) and visualized as outer rings around each node.

*Network centrality and bridge analysis*. After constructing the network, we computed network centrality indices to identify influential nodes. Following prior research, we excluded strength centrality due to its inability to account for negative correlations between variables (Robinaugh et al., 2016), and betweenness and closeness centrality due to their limited reliability (Bringmann et al., 2019). Instead, we adopted Expected Influence (EI) as a primary centrality measure, calculated by summing all edge weights connected to a given node. Higher EI values indicated that a node played a more central role in the network. Additionally, we examined bridge nodes, which facilitate connections between distinct network clusters and may serve as key intervention targets (Kaiser et al., 2021). To quantify this, we used Bridge Expected Influence (BEI), which sums the edge weights of a node linking to all nodes in other clusters (Jones et al., 2021). Given our focus on cross-cluster interactions, BEI serves as the primary metric for identifying nodes with the greatest potential to influence multiple network clusters.

*Network stability evaluation*. To assess the stability of the network, we examined both edge weight stability and node centrality stability. We employed a nonparametric bootstrap method (5,000 permutations) to evaluate the consistency of edge weights and computed the correlation stability coefficient (CS-coefficient) to measure the robustness of node centrality stability. Following established guidelines (Epskamp et al., 2018), we considered CS-coefficient values above 0.25 as indicative of sufficient reliability. Additionally, we conducted bootstrapped difference tests for edge weights and node centralities, with results visualized to facilitate interpretation.

*Age-stratified network analysis*. To examine developmental differences, we stratified the sample into three age groups (3-, 4-, and 5-year-olds) and constructed partial correlation networks for each group. For every network, we computed centrality indices (Expected Influence and Bridge Expected Influence) and assessed stability using the same bootstrapping procedures described earlier. We then conducted pairwise network comparisons across age groups to evaluate: general network strength (global connectivity), and structural invariance (difference in network topology). All analyses were performed in R version 4.4.1, utilizing the following packages: *mgm (for network estimation), bootnet (for stability assessment), qgraph (for visualization)* and *NetworkComparisonTest (for invariance testing)*. Statistical significance was set at *p* < 0.05 (two-tailed).

## 3 Result

### 3.1 Descriptive analysis

Sample Characteristics. Parents of 6,394 children participated in the study. Among the children evaluated, 3,372 were boys (52.7%) and 3,022 were girls (47.3%). The sample was stratified by age: 1,093 (17.1%) were 3-year-olds, 2,094 (32.7%) were 4-year-olds, and 3,207 (50.2%) were 5-year-olds. Geographically, 5,508 children (86.1%) resided in urban areas, while 886 (13.9%) lived in rural areas. Regarding kindergarten type, 2,821 (44.1%) attended public kindergartens, and 3,573 (55.9%) attended private kindergartens. The means and standard deviations of PDS and PRSS variables for each age group were presented in Table 1.

**Table 1:** PDS and PRSS in preschool children and age differences (*M* ± *SD*).

**Variables**	**Total (*N* = 6,394)**	**3 years (*n* = 1,093)**	**4 years (*n* = 2,094)**	**5 years (*n* = 3,207)**	***F***	***Post hoc* test**
**PDS:**						
Cognition:	4.03 ± 0.53	3.950 ± 0.53	4.000 ± 0.53	4.070 ± 0.54	24.29^***^	5 years > 4 years > 3 years
Emotion:	3.910 ± 0.49	3.900 ± 0.51	3.890 ± 0.50	3.930 ± 0.48	9.95^***^	5 years > 4 years = 3 years
Language:	3.820 ± 0.51	3.830 ± 0.51	3.840 ± 0.48	3.800 ± 0.52	5.47^**^	3 years = 4 years > 5 years
Art:	4.020 ± 0.56	4.010 ± 0.59	4.030 ± 0.57	4.020 ± 0.54	1.05	
Body:	3.560 ± 0.37	3.830 ± 0.40	3.530 ± 0.33	3.490 ± 0.35	312.29^***^	3 years > 4 years > 5 years
**PRSS:**						
Respect:	3.810 ± 0.48	3.810 ± 0.48	3.810 ± 0.47	3.810 ± 0.49	0.027	
Acceptance:	4.180 ± 0.53	4.200 ± 0.54	4.190 ± 0.52	4.160 ± 0.53	3.79^*^	3 years = 4 years > 5 years
Motivation:	3.930 ± 0.65	3.940 ± 0.64	3.940 ± 0.65	3.930 ± 0.65	0.21	
Democracy:	4.080 ± 0.59	4.110 ± 0.58	4.100 ± 0.59	4.060 ± 0.59	3.87^*^	3 years = 4 years > 5 years
Control:	2.770 ± 0.50	2.780 ± 0.51	2.780 ± 0.51	2.770 ± 0.49	0.46	
Protection:	3.400 ± 0.41	3.390 ± 0.40	3.400 ± 0.41	3.390 ± 0.41	0.99	

PDS, Preschool Development Scale; PRSS, Parental Rearing Style Scale; ^*^P < 0.05, ^**^P < 0.01, ^***^P < 0.001.

Age-related Differences in PDS scores. The PDS scores exhibited significant age-related differences across all dimensions except for Art. Key trends included: Cognitive Ability: increased progressively with age (3 < 4 < 5 years), supported by a strong main effect (*F* = 24.29, *p* < 0.001, partial η^2^ = 0.008); Emotional Competence: 5-year-olds scored significantly higher than both 3- and 4-year olds (*F* = 9.95, *p* < 0.001, partial η^2^ = 0.003); Language Ability: Contrary to other domains, 5-year-olds demonstrated lower language scores compared to younger groups (*F* = 5.47, *p* = 0.004, partial η^2^ = 0.002); Physical Ability: Decreased markedly across age groups (3 > 4 > 5 years), with the largest effect size observed (*F* = 312.29, *p* < 0.001, partial η^2^ = 0.106). Given the large sample size in this study (*N* = 6,394) and as noted by Wasserstein and Lazar (2016), statistically significant differences may reflect false positives; therefore, these findings should be interpreted with caution in light of their practical relevance.

Age-related differences in PRSS scores. Only the Acceptance and Democracy dimensions exhibited minor but statistically significant age-related differences. Specifically, 5-year-olds scored slightly lower than 3- and 4-year-olds in both dimensions (Acceptance: *F* = 3.79, *p* < 0.05, partial η^2^ = 0.001; Democracy: *F* = 3.87, *p* < 0.05, partial η^2^ = 0.001). Overall, parents predominantly reported employing parenting styles emphasizing Respect, Acceptance, Motivation, Democracy, Control, and Protection, suggesting a positive trend across all dimensions. Furthermore, correlation analysis revealed a moderate-to-strong association (*r* = 0.54, *p* < 0.001) between parenting style and children's physical and mental development, indicating that more adaptive parenting practices were linked to better developmental outcomes (see Table 2). The correlation heatmaps illustrating age-related changes among specific dimensions are presented in Supplementary Figures S1–S4.

**Table 2:** Matrix of correlations among variables.

**Variables**				
1.Gender	1			
2. Age	−0.005	1		
3. PDS	0.112^***^	−0.026^***^	1	
4. PRSS	0.053^***^	−0.022	0.540^***^	1

PDS, Preschool Development Scale; PRSS, Parental Rearing Style Scale; ^***^P < 0.001.

### 3.2 General network analysis

**Figure 1:**
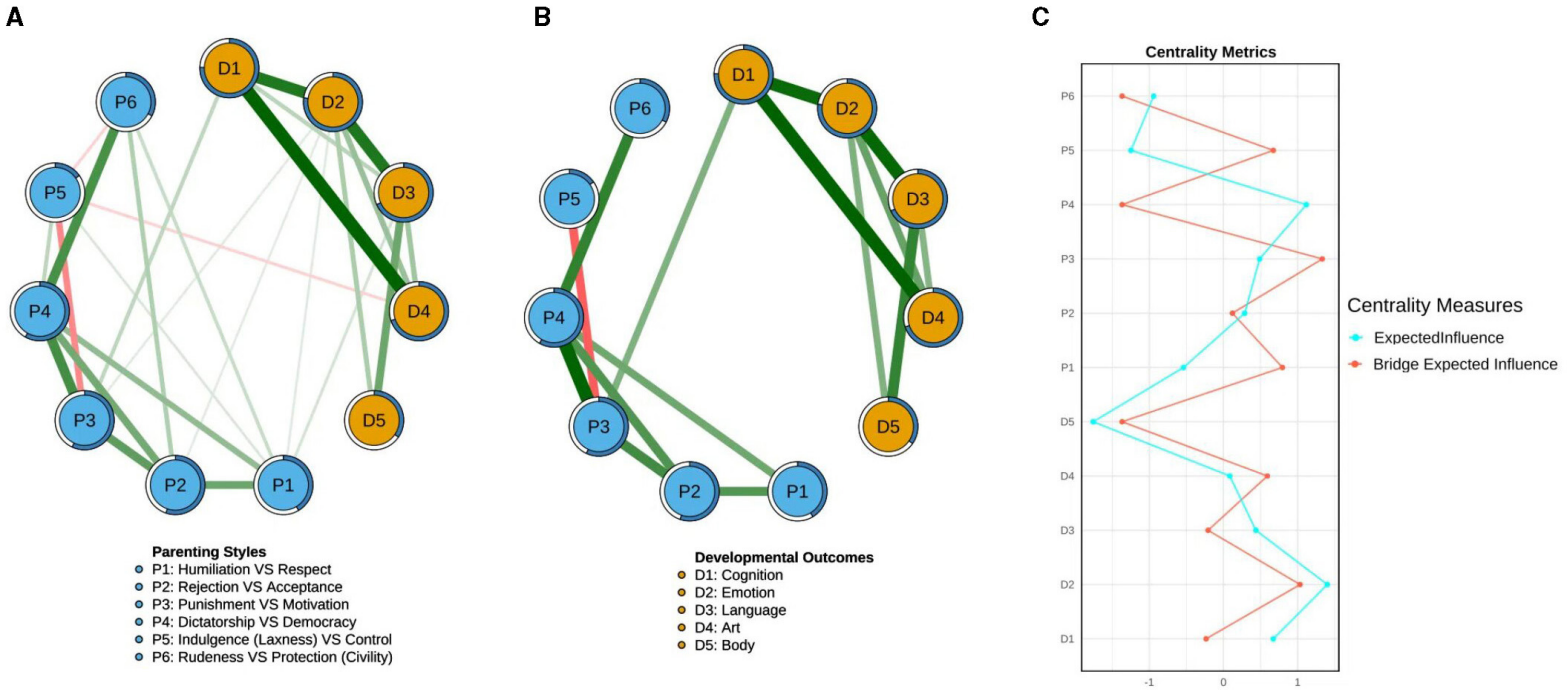
The parenting-development network structure of preschool children **(A)**, the network structure after filtering out edges with weights below 0.1 **(B)**, and centrality and bridge centrality metrics **(C)**. In the **(A, B)** diagrams, light blue nodes represent factors of parenting style, while orange nodes represent factors of physical and mental development. The thickness of an edge indicates the strength of the correlation between two nodes, with green edges representing positive correlations and red edges representing negative correlations. The rings around the nodes represent predictability. In the **(C)** diagram, a higher Expected Influence indicates that a node has stronger connections with other nodes, while a higher Bridge Expected Influence suggests that a node within a cluster has stronger connections to nodes in other clusters.

**Figure 2:**
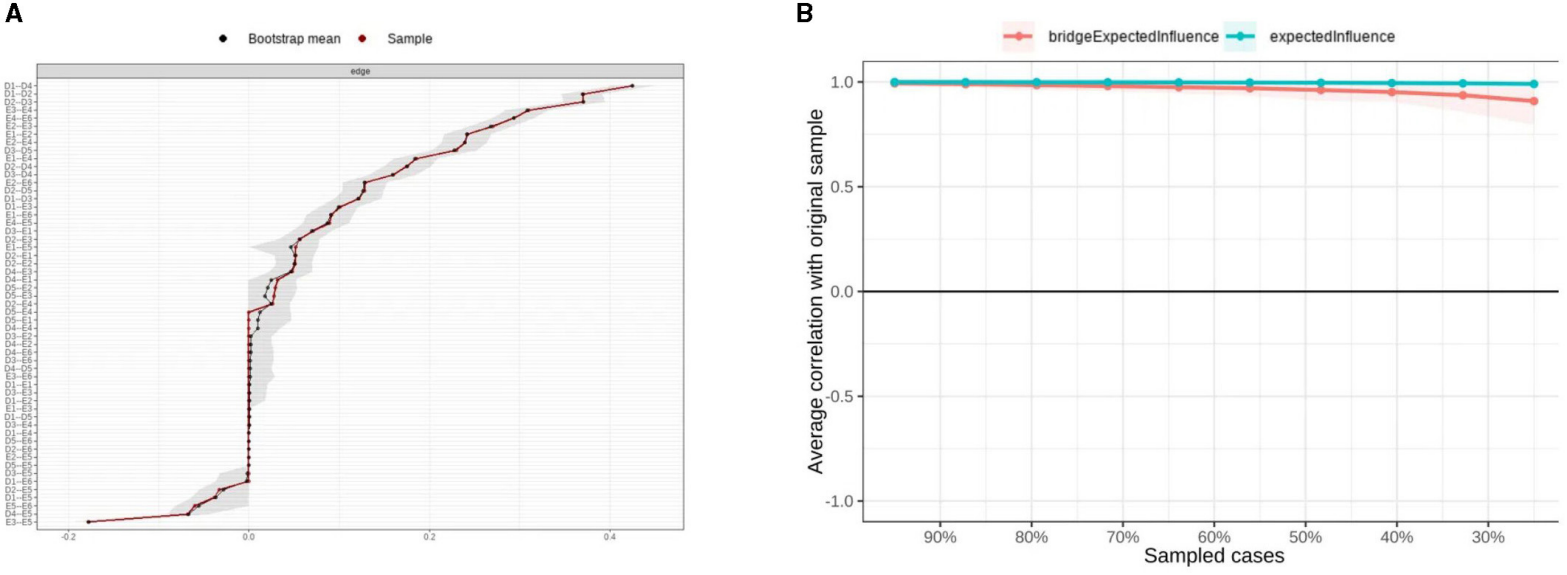
Edge weight accuracy test of the network using the non-parametric bootstrapping method **(A)** and stability of centrality metrics using the case-drop bootstrapping method **(B)**. In the **(A)** diagram, the sample edge weight is illustrated by a red line, while the bootstrap mean edge weight is illustrated by a black line. The gray area represents the bootstrapped confidence intervals, with narrower confidence intervals indicating more reliable accuracy. In the **(B)** diagram, the line represents the averaged correlation between the centrality metrics of the original sample and the subsamples.

The parenting-development network of preschool children is illustrated in Figure 1A. The strongest within-cluster connections were observed between: Cognition and Art (D1-D4), Emotion and Language (D2-D3), and Cognition and Emotion (D1-D2). Among cross-cluster connections, the strongest link was between Motivation and Cognition (P3-D1) (see Figure 1B, where edges with weights < 0.1 were filtered out). The general centrality indices are shown in Figure 1C. Bootstrap analysis (95% CIs) confirmed the reliability of edge weights (see Figure 2A), with narrow confidence intervals indicating estimation precision.

Centrality and Bridging Effects. Expected Influence (EI) centrality identified Emotion (D2) as the most central node, followed by Democracy (P4), highlighting their pivotal roles in the network. Bridge Expected Influence (BEI) analysis revealed Motivation (P3) and Emotion (D2) as the strongest bridge nodes, suggesting: Motivation (P3) had the strongest ties to the development cluster; Emotion (D2) was most closely linked to the parenting style cluster. Both Expected Influence and Bridge Expected Influence demonstrated high stability (CS coefficient = 0.75; see Figure 2B). Average node predictability was 53.3%, indicating that over half of the variance in a node's activity could be explained by its neighboring nodes.

### 3.3 Age-related network analysis

**Figure 3:**
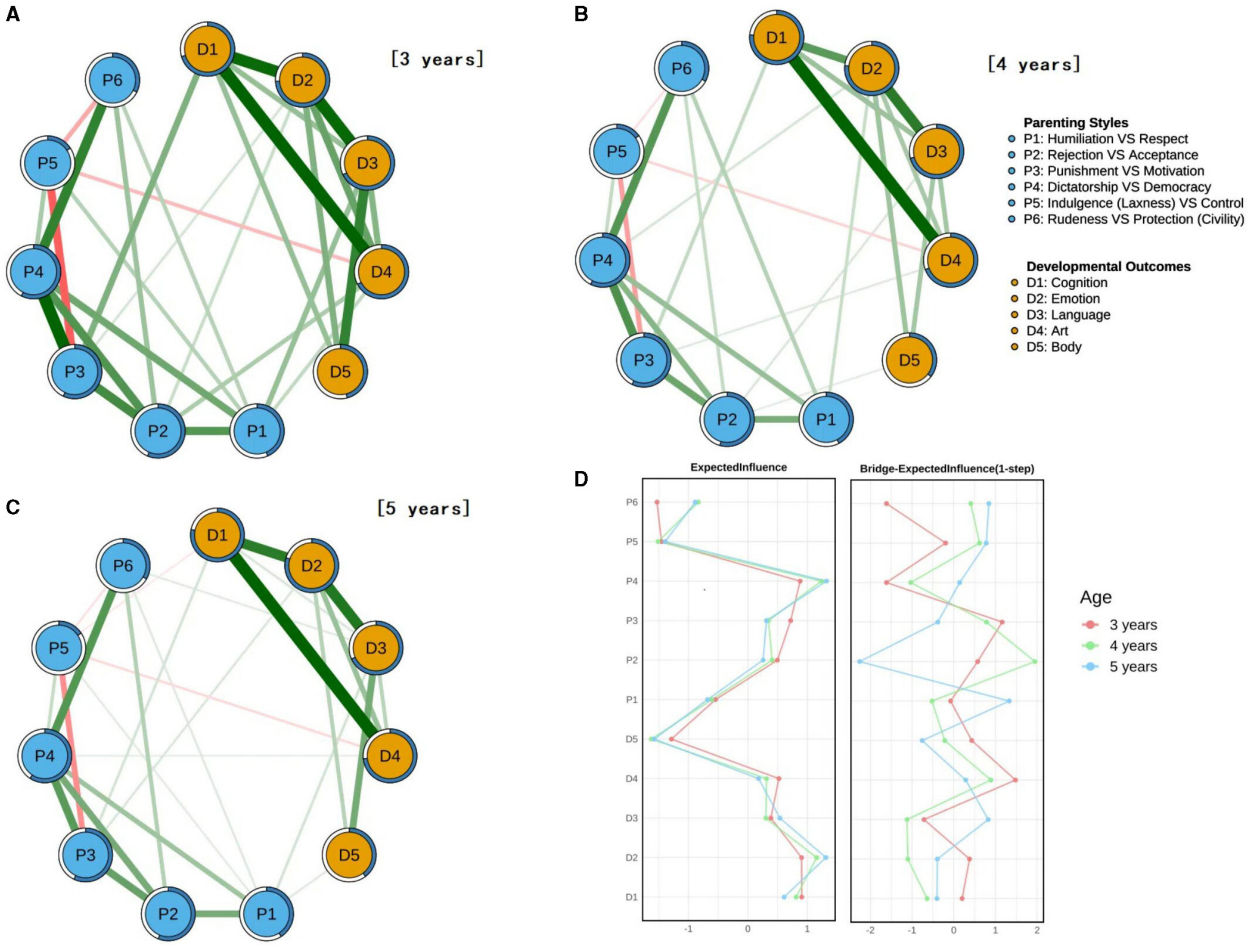
Parenting-development network structures and centrality metrics across different age groups. **(A–C)** represent the network structures for the 3-, 4-, and 5-year-old groups, respectively. Light blue nodes indicate parenting style factors, while orange nodes represent physical and mental development factors. Edge thickness reflects the strength of correlation between two nodes. Green edges denote positive correlations, whereas red edges indicate negative correlations. Rings around nodes represent predictability. **(D)** illustrates the centrality metrics for the three age groups. Higher Expected Influence values indicate stronger connections between a node and others. Higher Bridge Expected Influence values suggest a stronger connection between a node and those in different clusters.

The age-stratified network structures are presented in Figure 3, with filtered network visualizations provided in Supplementary Figure S5. Consistent with the general network, the strongest within- cluster connections across all age groups were observed between: Cognition and Art (D1-D4), Emotion and Language (D2-D3), and Cognition and Emotion (D1-D2). Below we present detailed network characteristics for each age cohort.

Three-year-olds Cohort. The network analysis revealed Motivation (P3) and Cognition (D1) as exhibiting the strongest cross- cluster connection. Centrality analysis identified Cognition (D1) and Emotion (D2) as nodes with the highest Expected Influence (EI), while Art (D4) and Motivation (P3) demonstrated the greatest bridge expected influence (BEI).

Four-year-olds Cohort. Similar to the 3-year-olds network, the strongest cross-cluster connection remained between Motivation (P3) and Cognition (D1). However, centrality patterns showed notable developmental shifts: Democracy (P4) and Emotion (D2) emerged as the most influential nodes (EI), while Acceptance (P2) and Art (D4) showed the strongest bridge properties (BEI). This pattern suggests an increasing role of Acceptance-related parenting behaviors in this age group.

Five-year-olds Cohort. The network structure showed distinct developmental progression, with Respect (P1) and Language (D3) forming the strongest cross-cluster connection. Centrality analysis revealed Democracy (P4) and Emotion (D2) as maintaining the highest EI, while Respect (P1) and Language (D3) exhibited the greatest BEI. This pattern indicates a maturation of socio-communicative networks in the oldest cohort.

Age-related Shifts in Network Centrality and Stability. Notably, Respect (P1) emerged as the most influential bridge node in the 5-year-old cohort, surpassing other parenting styles—a marked shift from younger age groups. Conversely, Acceptance (P2) exhibited a significant decline in bridging influence. Bootstrap analysis confirmed the robustness of edge weight estimates across all networks (Supplementary Figures S6–S8). Centrality stability metrics further supported the reliability of our findings: EI demonstrated strong stability (CS coefficients = 0.75); BEI showed acceptable stability (CS coefficients = 0.52). Following established criteria (Epskamp et al., 2018), CS coefficients > 0.5 indicate good stability, confirming the reliability of centrality metrics in all three age-stratified networks (Supplementary Figures S9–S11).

Age-related Differences in Network Structure and Connectivity. To assess the influence of age on parenting-development network dynamics, we conducted pairwise comparisons across age groups, evaluating both global network strength and structural invariance. The structural invariance revealed significant differences: 3- and 4-year-olds (*M* = 0.138, *p* < 0.01), 3- and 5-year-olds (*M* = 0.148, *p* < 0.01), and 4- and 5-year-olds (*M* = 0.119, *p* < 0.01). Despite age-related shifts in connection patterns, the overall network connectivity remained stable (*p* > 0.05), suggesting potential compensatory pathways between parenting styles and preschool child development (Waters et al., 2019; Sun et al., 2024). As to edge-specific changes in cross- cluster connections, further edge-weight analysis (see Table 3) identified key developmental shifts: respect-Language (P1-D3) decreased sharply from 0.14 (3-year-olds) to 0.01 (4-year-olds), then partially recovered to 0.08 (5-year-olds, *p* < 0.05); Acceptance-Language (P2-D3) emerged at 0.07 (4-year-olds) after being absent at age 3, then dropped to 0.01 (5-year-olds, *p* = 0.028). These findings indicate that parenting-language associations reorganize dynamically across early childhood, with Respect (P1) losing early prominence while Acceptance shows a transient influence at age 4.

**Table 3:** Age-related changes in the characteristics of parenting styles and physical and mental development of preschool children.

**Edge connection**	**3 years**	**4 years**	**5 years**	***P-*value**	**Change**
D1-D2	0.325	0.301	0.392	0.037	5 years > 4 years = 3 years
D1-D3	0.138	0.183	0.069	0.027	3 years = 4 years > 5 years
D1-D4	0.339	0.467	0.467	0.018	5 years = 4 years > 3 years
D1-D5	0.139	0.028	0.032	0.037	3 years > 4 years = 5 years
P1-D3	0.137	0.013	0.078	0.018	3 years > 4 years =5 years
P2-D3	0	0.065	0.008	0.028	4 years > 3 years = 5 years

No edges were found to differ across all three age groups in pairwise comparisons. The p-values indicate the significance of differences between the two networks connected by the “>” symbol.

## 4 Discussion

This study employed network analysis to delineate the structure of parenting–developmental network patterns in Chinese preschool children and to explore age-related variations. By examining centrality, bridge centrality, and cross-cluster connections, we identified dynamic shifts in how parenting styles interact with developmental outcomes across early childhood. Our findings extend beyond traditional perspectives, which suggest that positive parenting styles uniformly benefit physical and mental development. Instead, we reveal age-specific patterns of influence, suggesting that the relative importance of different parenting strategies, such as Respect (P1) and Acceptance (P2), varies as children grow. For instance, the increasing centrality of Respect in older preschoolers and the transient role of Acceptance at age 4 highlight the need for developmentally tailored parenting approaches.

### 4.1 The motivation–cognition–emotion connection pattern in the general network

Our network analysis identified Emotion (D2) as the most central and influential node in preschool children's physical and mental development. It demonstrated the highest Expected Influence and Bridge Expected Influence, indicating its dual role as both a primary mediator between parenting styles and developmental outcomes, and as a critical bridge connecting multiple developmental domains. This centrality suggests that emotional development serves as a foundational element integrating various aspects of early childhood growth, with parenting practices potentially exerting their effects largely through emotional pathways.

Among parenting styles, Motivation (P3) emerged as the most influential node in cross-domain connections, exhibiting the highest Bridge Expected Influence. While Democracy (P4) showed the greatest overall centrality, its lack of direct connections to developmental nodes suggests its effects may be indirectly mediated through motivational mechanisms. This finding highlights motivation's crucial role as a bridging factor that translates parenting behaviors into developmental outcomes. The strongest cross-cluster connection between Motivation and Cognition (P3-D1) further supports this interpretation, suggesting a key pathway through which parenting may influence cognitive development.

The network construct revealed particularly close connections between Cognition (D1) and both Art (D4) and Emotion (D2). This pattern likely reflects conceptual overlap in our measurement instruments, where items assessing artistic ability (e.g., enjoying music games with adults and peers, experiencing melodies and emotions in music) inherently incorporate emotional components. These connections suggest that cognitive development in preschoolers is deeply intertwined with both artistic engagement and emotional processing, forming an integrated developmental triad. Our finding aligns with established neurodevelopmental evidence. The rapid maturation of the amygdala–ventromedial prefrontal cortex (vmPFC) circuit during preschool years provides a neural substrate for the observed cognition-emotion integration (Tottenham and Gabard-Durnam, 2017). Furthermore, research demonstrates that external stimuli and rewards facilitate the development of higher-order cognitive networks through this pathway (Salzwedel et al., 2019), offering a mechanistic explanation for how motivational parenting behaviors might simultaneously enhance cognitive and emotional abilities. Behavioral studies provide additional support for our network findings. Preschool children consistently show preferences for direct motivational experiences, including verbal praise from caregivers and tangible rewards (Frankel et al., 2012; Luo et al., 2021). These motivational inputs appear to trigger a synergistic competencies in language acquisition and social skills (Kam et al., 2004; Riggs et al., 2006). This evidence strengthens our interpretation of motivation's bridging role in the parenting-development network.

Theoretical integration of our findings suggests a cyclical mediation model consistent with Pekrun et al.'s (2023) achievement emotion theory. We propose that multiple parenting styles may be associated with child development through a sequential process: parenting behaviors—enhanced motivation - [cognition-emotion interaction] - developmental outcomes. This developmental pathway might reflect our primary findings and potentially account for secondary patterns in the network, such as the association between Respect and Language development (P1–D3). However, considering that many studies have found children's behaviors can also shape parenting styles (Wang and Gai, 2024), this pathway may also be reversed or dynamically bidirectional—a possibility we further explore in the Age-Specific Network section.

### 4.2 Age-specific network structures and developmental transitions

Our network analysis revealed significant age-related changes in parenting-development connections, characterized by a gradual shift from direct to indirect influence patterns. While network strength invariance tests showed no significant differences in connection density across age groups (3-, 4-, and 5-year-olds), network structure invariance tests indicated substantial topological reorganization (van Borkulo et al., 2023). The most striking transition involved shifting central parenting factors: Motivation (P3) served as the primary bridge node at age 3, was replaced by Acceptance (P2) at age 4, and ultimately by Respect (P1) at age 5. This progression might reflect children's developmental trajectory from relying on material incentives to valuing behavioral acceptance (Rohner, 2021) and eventually seeking emotional respect (Doh et al., 2016). Corresponding changes emerged in cross-domain connections, with the dominant Motivational-Cognitive (P3-D1) links in younger children giving way to Respect-Language (P1-D3) connections by age 5. Developmental hubs similarly transitioned from artistic engagement (D4) to language skills (D3), mirroring increasing verbal mediation in parent-child interaction. These patterns align with Theory of Mind development, as growing mentalizing capacity enables children to infer parental intentions and internalize respect-based interaction (Minty, 2000). These findings also support a compensatory socialization model, where kindergarten exposure provides alternative social models that help children reconstruct parent-child dynamics around acceptance and respect norms (Morawska et al., 2019). Together, these results suggest that the evolving cognitive and social capacities of preschool children may interact with and contribute to changes in the structure of parenting-development networks.

Our edge-weight comparisons further elucidated the nuanced developmental shifts in parenting-development networks across ages 3-5. Notably, the connections between Cognition and Emotion (D1-D2) and Cognition and Art (D1-D4) strengthened progressively with age, while the direct link between Cognition and Language (D1-D3) weakened, a pattern consistent with the affective-behavioral-cognitive dynamic model (Greenberg and Kusché, 2006). This might suggest a developmental transition where emotion gradually supplants cognition as the primary mediator between cognition and language development, a finding corroborated by intervention research demonstrating emotion's pivotal role in scaffolding early language acquisition (Bierman et al., 2010; Kam et al., 2004). The observed cross-cluster changes highlighted distinctive features of age-specific networks, with the robust Cognition–Art (D1–D4) connection and increasing Bridge Expected Influence underscoring art's critical role during ages 3 to 4. Existing research indicates that artistic activities involve the complex processing of emotional information (Vuoskoski and Eerola, 2011), which not only promotes a dynamic interplay between cognitive and emotional functions but also shapes openness personality traits characterized by high imagination and rich emotionality from early life, subsequently influencing performance in adolescence and adulthood (Ruth et al., 2023). At this developmental stage, children exhibit heightened sensitivity to emotionally evocative artistic stimuli—such as singing, dancing, and painting—which aligns with evidence that artistic engagement facilitates internal reinforcement of holistic development (Menzer, 2015). These findings collectively underscore how dynamic reweighting of network connections reflects children's evolving developmental priorities, with emotional and artistic domains gaining prominence as mediators between core cognitive and linguistic capacities during the preschool years. The identified patterns provide actionable insights for designing developmentally targeted interventions that leverage these naturally occurring transitions in children's learning architectures.

The unique presence of the Acceptance and Language (P2-D3) connection in the 4-year-olds network highlights a critical developmental phase where children begin evaluating their behavior through environmental exploration and parental reactions (Orenstein and Lewis, 2024). Unlike 3-year-olds, who struggle with false belief tasks and lack full perspective-taking ability (Stipek et al., 1992), and 5-year-olds, who have developed a more stable self-concept with higher expectations (Erikson, 1951), 4-year-olds appear to rely on parental acceptance as a scaffold for language development. Meanwhile, the Respect and Language (P1-D3) connection, though weakened with age, remained the strongest cross-cluster link, surpassing Motivation-Cognition, supporting a shift in parenting patterns. By age 5, as children enter a rapid language growth phase and possess stronger cognitive-emotional foundations, respect becomes more crucial in parent-child interactions (Doh et al., 2016). These findings suggest that parenting strategies should adapt to developmental stages: emphasizing responsive acceptance for 4-year-olds to support self-regulation and language development, while transitioning to respect-based dialogue for 5-year-olds to align with their growing autonomy and social-cognitive maturity.

Parenting strategies should evolve to align with child's developmental stages, emphasizing motivation-based approaches for preschoolers (Morris et al., 2017). Rather than relying on material rewards, parents can use high-emotional-arousal strategies, such as praise, hugs, or role-playing games (Yazar and Tuzgöl Dost, 2024), alongside verbal guidance to nurture intrinsic motivation (Bjørk et al., 2020). For 3- to 4-year-olds, fostering motivation through artistic activities—especially those embedded within the family environment as significant external stimuli—can strengthen the links between cognition and emotion, thereby supporting holistic development, the formation of openness personality traits, and overall socialization (Kreutz and Feldhaus, 2018). As children approach age 4–5, parents should increase their tolerance for exploratory behaviors, replacing punishment with constructive communication; accepting mistakes as part of the learning process predicts positive outcomes (Marceau et al., 2013). By age 5 and beyond, a “psychological transition” becomes key: prioritizing respect, autonomy in decision-making (e.g., clothing, food choices), and active listening helps cultivate social and language skills. This progression, from motivation-driven scaffolding to respect-based autonomy, ensures parenting adapts to children's growing cognitive, emotional, and social needs.

## 5 Limitations and future directions

Several limitations of the present study warrant discussion. First, the PDS and PRSS questionnaires in this study were completed independently by parents of preschool children. Given the competitive cultural context in China, responses may be subject to social desirability bias (Chen et al., 2015). Prior research suggests that Chinese parents report higher levels of socially desirable responding than their Western counterparts, potentially leading to overestimation of children's abilities (e.g., academic-related cognition) and underreporting of negative parenting practices (e.g., physical punishment) (Bornstein et al., 2015). These factors should be considered when interpreting the data. Second, while we focused on dominant network connections, weaker but potentially meaningful patterns, such as the link between Control and Art (P5-D4), were not explored in depth. Future research could employ mediation models to examine whether these connections operate through indirect pathways. Third, the cross-sectional design precludes causal inferences; longitudinal studies are needed to clarify how age shapes dynamic changes in parenting-development networks (Liang et al., 2025). Last, our network comparisons may inflate Type I error rates. Advanced methods like network embedding (Grover and Leskovec, 2016) could mitigate this risk by capturing higher-order structural similarities across developmental stages. Addressing these limitations will be critical for refining tailored parenting strategies and validating the broader network architecture of child development.

## Conclusion

This study investigated the specific and age-related associations between parenting styles and developmental outcomes in preschool children using network analysis, revealing Motivation and Emotion as central bridging nodes, interconnected via Cognition. These results suggest that children may develop intrinsic motivation through a “Motivation–Cognition–Emotion” pathway. Age-group analyses revealed distinct patterns: 3- to 4-year-olds relied heavily on art-based activities; 4-year-olds uniquely benefited from Acceptance; and 5-year-olds demonstrated stronger associations with Respect and Language, consistent with their advancing social and communicative needs. These findings offer practical guidance for parents by supporting the use of age-tailored strategies to optimize children's cognitive and emotional development.

## Data Availability

**T**he datasets presented in this study can be found in online repositories. The names of the repository/repositories and accession number(s) can be found below: https://www.scidb.cn/en/s/nQnaUf.
