# Supplementary material for: Parenting styles and preschool children's development: from network analysis perspective
Source: Front Psychol. 2025 Jul 11;16:1624317. doi: 10.3389/fpsyg.2025.1624317 (PMC12289661; doi:10.3389/fpsyg.2025.1624317)
Supplement: Supplementary file 1 [file Supplementary_file_1.docx]

Supplementary Material

# Supplementary Figures


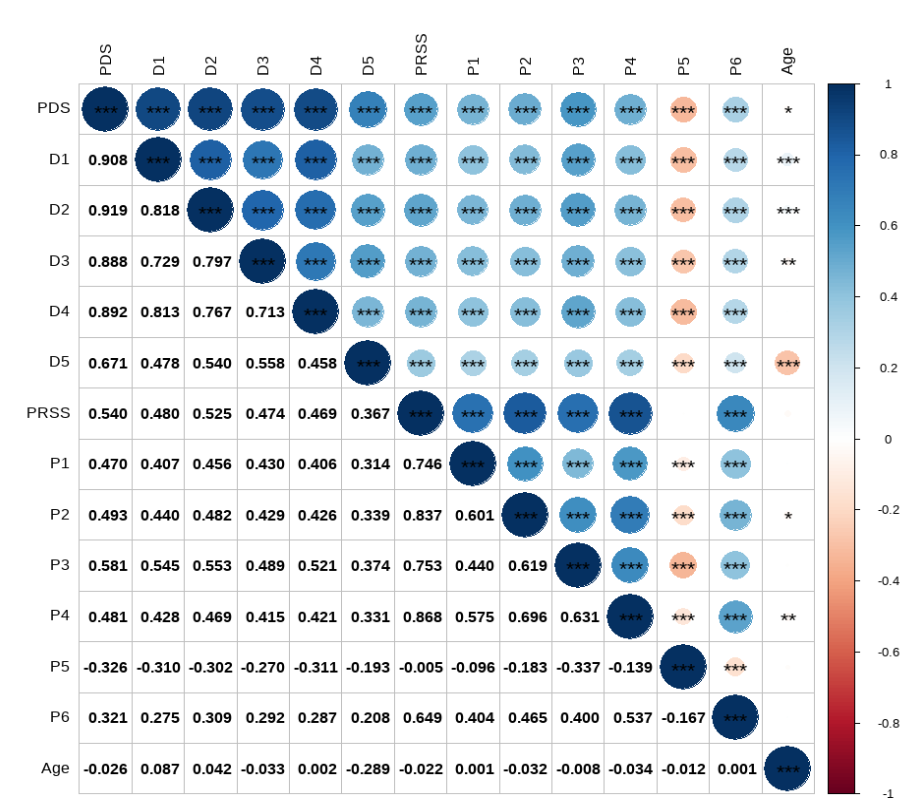


Supplementary Figure S1. Correlation heatmap of PDS, PRSS, and their subdimensions across age in the overall sample

Note: The heatmap shows correlations between numeric variables. The upper triangle displays variable names and significance levels (**P* < 0.05, ***P* < 0.01, ****P* < 0.001), while the lower triangle shows correlation coefficients to three decimals. Blue indicates positive and red negative correlations. PDS, Preschool Development Scale; PRSS, Parental Rearing Style Scale; D1 = cognition, D2 = emotion, D3 = language, D4 = art, D5 = body; P1 = respect, P2 = acceptance, P3 = motivation, P4 = democracy, P5 = control, P6 = protection.


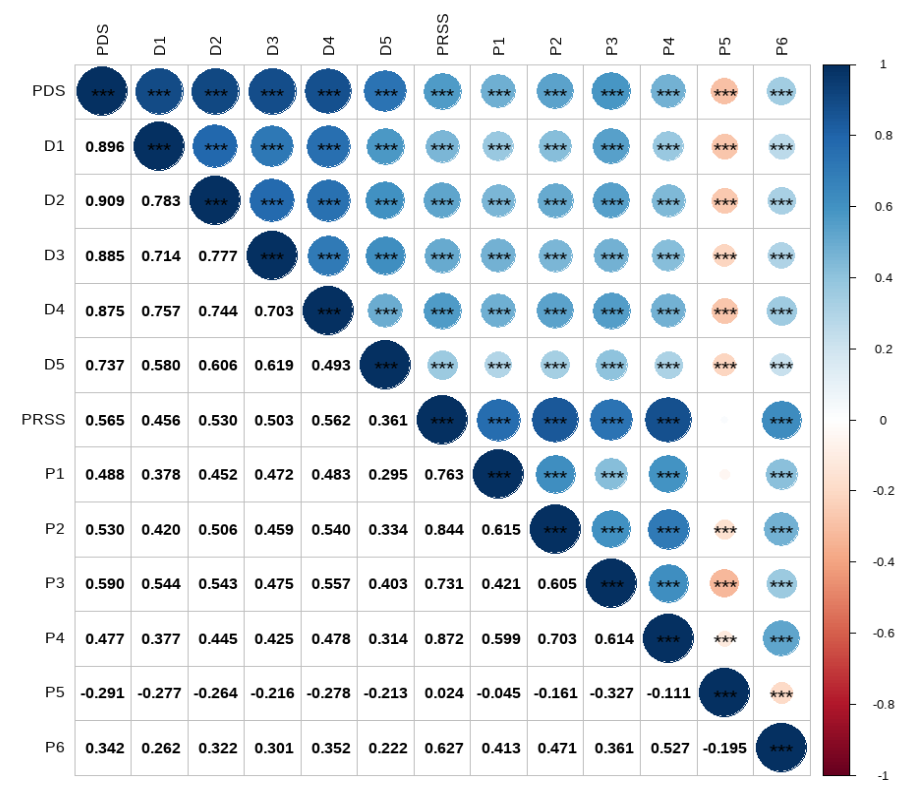


Supplementary Figure S2. Correlation heatmap of PDS, PRSS, and their subdimensions in 3-year-old children

Note: The heatmap shows correlations between numeric variables. The upper triangle displays variable names and significance levels (****P* < 0.001), while the lower triangle shows correlation coefficients to three decimals. Blue indicates positive and red negative correlations. PDS, Preschool Development Scale; PRSS, Parental Rearing Style Scale; D1 = cognition, D2 = emotion, D3 = language, D4 = art, D5 = body; P1 = respect, P2 = acceptance, P3 = motivation, P4 = democracy, P5 = control, P6 = protection.


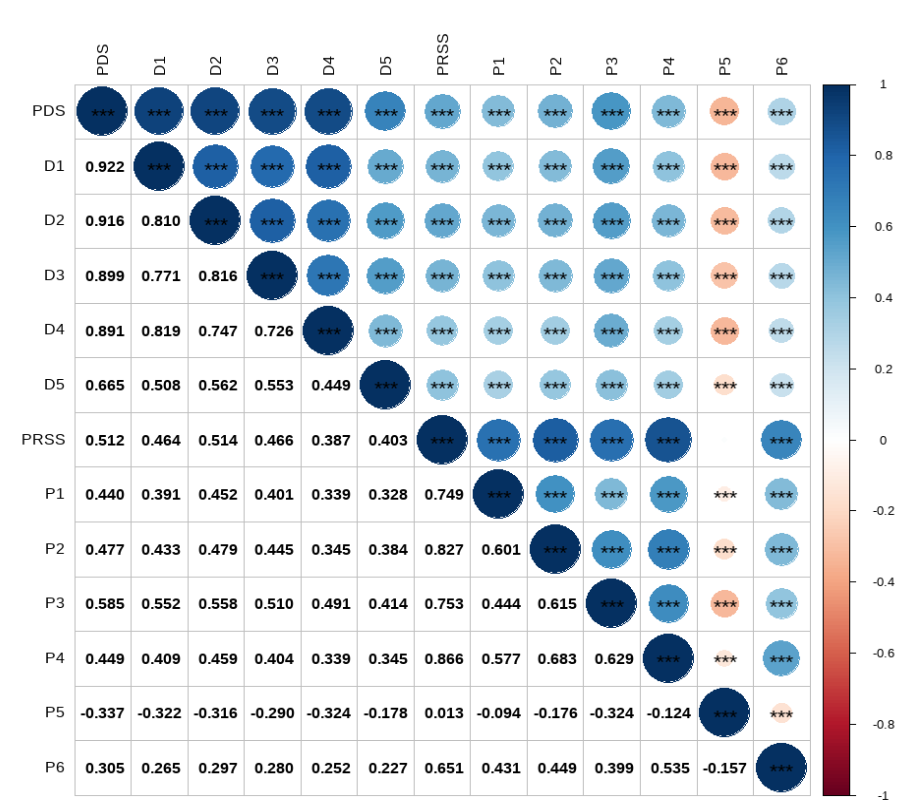


Supplementary Figure S3. Correlation heatmap of PDS, PRSS, and their subdimensions in 4-year-old children

Note: The heatmap shows correlations between numeric variables. The upper triangle displays variable names and significance levels (****P* < 0.001), while the lower triangle shows correlation coefficients to three decimals. Blue indicates positive and red negative correlations. PDS, Preschool Development Scale; PRSS, Parental Rearing Style Scale; D1 = cognition, D2 = emotion, D3 = language, D4 = art, D5 = body; P1 = respect, P2 = acceptance, P3 = motivation, P4 = democracy, P5 = control, P6 = protection.


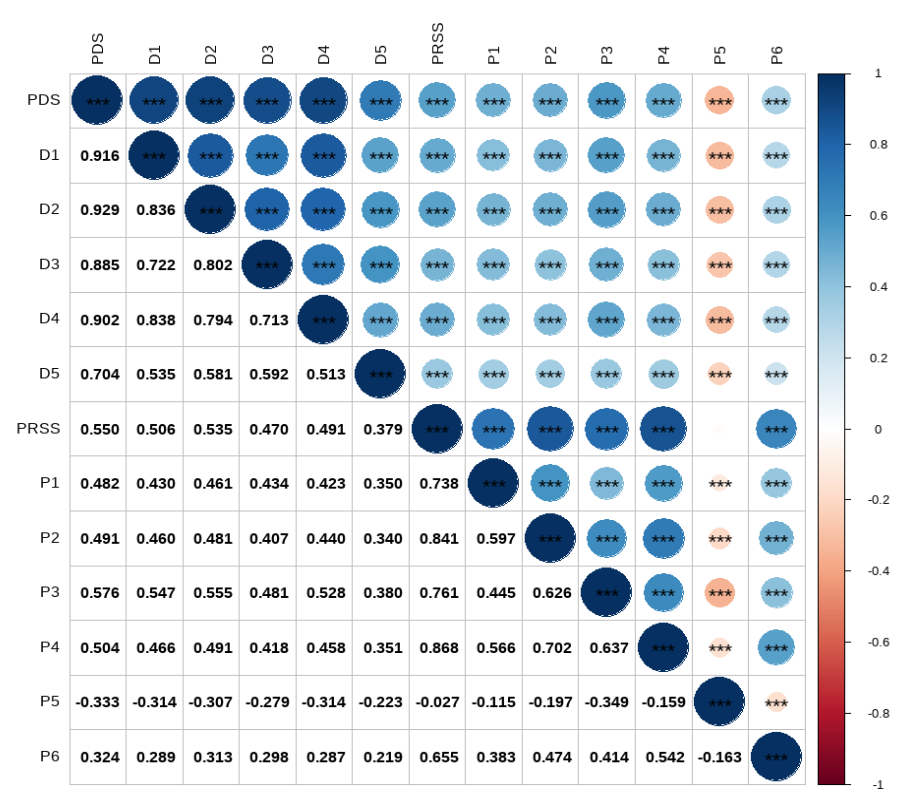


Supplementary Figure S4. Correlation heatmap of PDS, PRSS, and their subdimensions in 5-year-old children

Note: The heatmap shows correlations between numeric variables. The upper triangle displays variable names and significance levels (****P* < 0.001), while the lower triangle shows correlation coefficients to three decimals. Blue indicates positive and red negative correlations. PDS, Preschool Development Scale; PRSS, Parental Rearing Style Scale; D1 = cognition, D2 = emotion, D3 = language, D4 = art, D5 = body; P1 = respect, P2 = acceptance, P3 = motivation, P4 = democracy, P5 = control, P6 = protection.


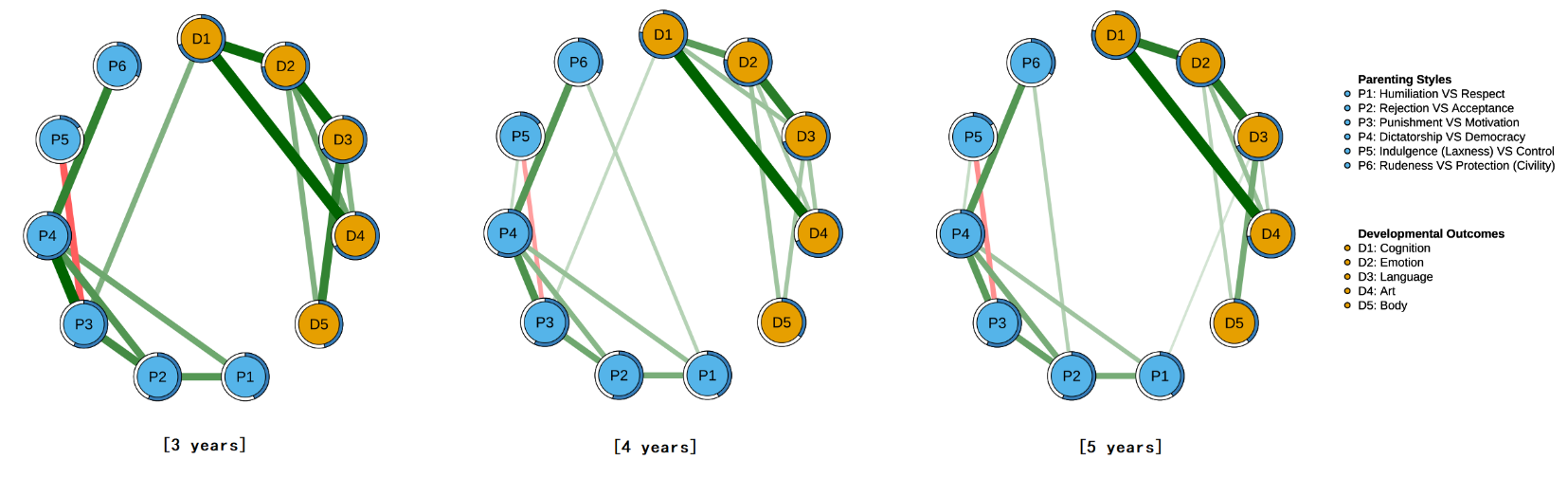


Supplementary Figure S5. Network structures of preschool children's parenting-development across different age groups (filtered)

Note: **(A)**, **(B)**, and **(C)** represent the network structures for the 3-year-old, 4-year-old, and 5-year-old groups, respectively. Light blue nodes represent factors of parenting style, while orange nodes represent factors of physical and mental development. The thickness of an edge indicates the degree of correlation between two nodes. Green edges represent positive correlations, while red edges represent negative correlations. The rings around the nodes indicate predictability.


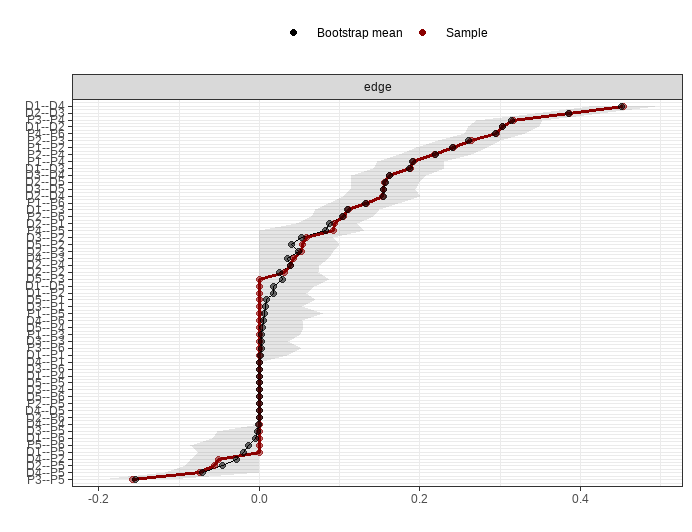


Supplementary Figure S6. Testing Edge-Weight Accuracy of the 3-Year-Old Network Using Non-Parametric Bootstrap Method

Note: The sample edge-weight is illustrated by the red line, and the bootstrap mean edge-weight is illustrated by the black line. The grey area represents the bootstrapped confidence intervals (CIs). As shown in the figure, the red and black lines almost overlap, and the 95% CI of the edge weights is relatively small, indicating that the edge weights in the constructed network are accurate. D1 = cognition, D2 = emotion, D3 = language, D4 = art, D5 = body; P1 = respect, P2 = acceptance, P3 = motivation, P4 = democracy, P5 = control, P6 = protection.


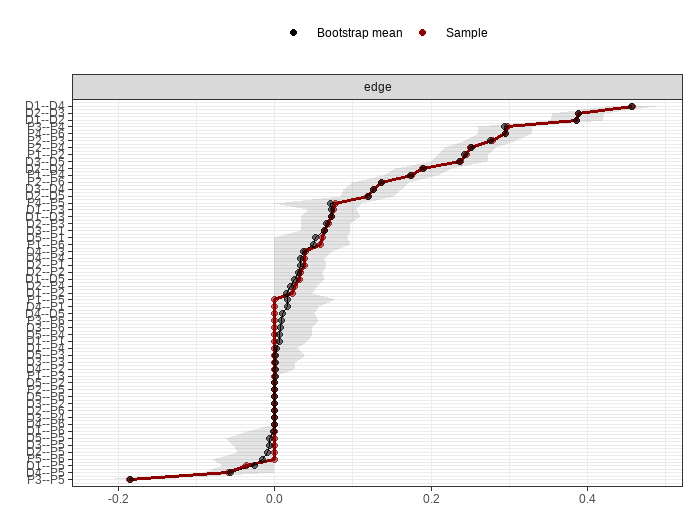


Supplementary Figure S7. Testing Edge-Weight Accuracy of the 4-Year-Old Network Using Non-Parametric Bootstrap Method

Note: The sample edge-weight is illustrated by the red line, and the bootstrap mean edge-weight is illustrated by the black line. The grey area represents the bootstrapped confidence intervals (CIs). As shown in the figure, the red and black lines almost overlap, and the 95% CI of the edge weights is relatively small, indicating that the edge weights in the constructed network are accurate. D1 = cognition, D2 = emotion, D3 = language, D4 = art, D5 = body; P1 = respect, P2 = acceptance, P3 = motivation, P4 = democracy, P5 = control, P6 = protection.


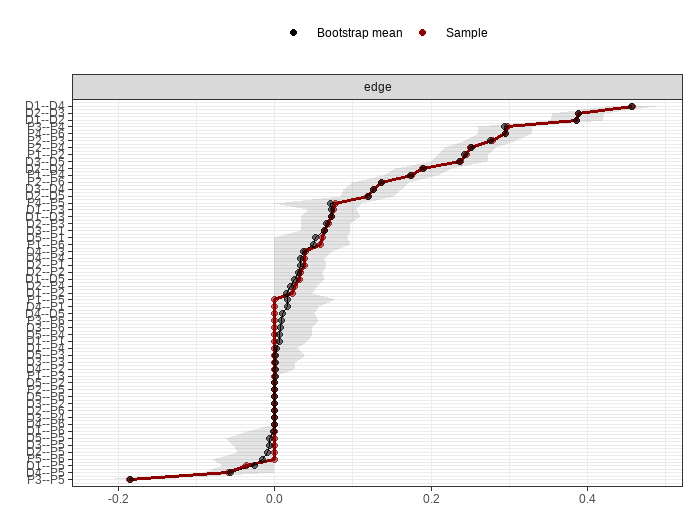


Supplementary Figure S8. Testing Edge-Weight Accuracy of the 5-Year-Old Network Using Non-Parametric Bootstrap Method

Note: The sample edge-weight is illustrated by the red line, and the bootstrap mean edge-weight is illustrated by the black line. The grey area represents the bootstrapped confidence intervals (CIs). As shown in the figure, the red and black lines almost overlap, and the 95% CI of the edge weights is relatively small, indicating that the edge weights in the constructed network are accurate. D1 = cognition, D2 = emotion, D3 = language, D4 = art, D5 = body; P1 = respect, P2 = acceptance, P3 = motivation, P4 = democracy, P5 = control, P6 = protection.


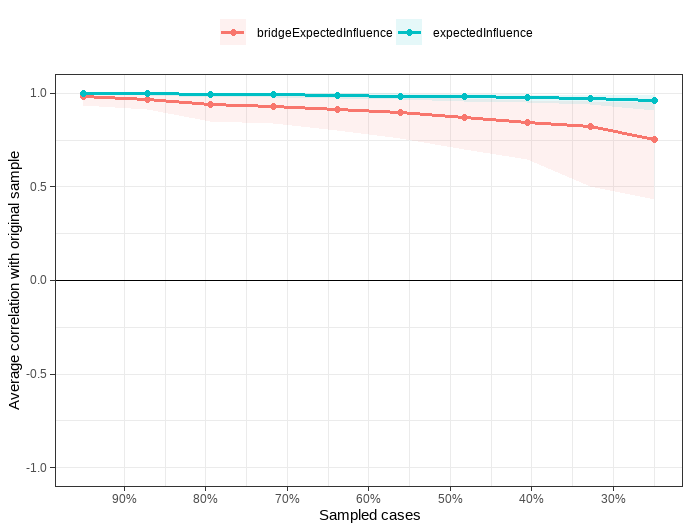


Supplementary Figure S9. Stability of Node Expected Influence and Bridge Expected Influence in the 3-Year-Old Network Using the Case-Drop Bootstrapping Method

Note: The red/blue line represents the averaged correlation between node expected influences in the original sample and subsamples. The red/blue shaded area indicates the range from the 2.5th quantile to the 97.5th quantile. The CS coefficient for Expected Influence is 0.75, and for Bridge Expected Influence, it is 0.517.


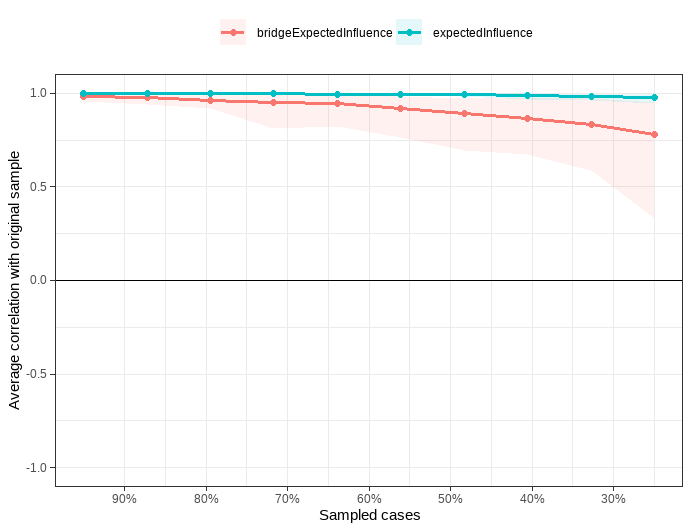


Supplementary Figure S10. Stability of Node Expected Influence and Bridge Expected Influence in the 4-Year-Old Network Using the Case-Drop Bootstrapping Method

Note: The red/blue line represents the averaged correlation between node expected influences in the original sample and subsamples. The red/blue shaded area indicates the range from the 2.5th quantile to the 97.5th quantile. The CS coefficient for Expected Influence is 0.75, and for Bridge Expected Influence, it is 0.517.


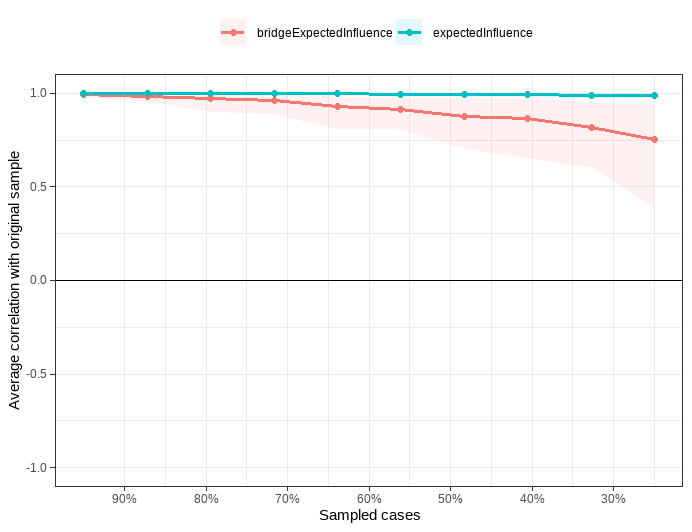


Supplementary Figure S11. Stability of Node Expected Influence and Bridge Expected Influence in the 5-Year-Old Network Using the Case-Drop Bootstrapping Method

Note: The red/blue line represents the averaged correlation between node expected influences in the original sample and subsamples. The red/blue shaded area indicates the range from the 2.5th quantile to the 97.5th quantile. The CS coefficient for Expected Influence is 0.75, and for Bridge Expected Influence, it is 0.517.
